# Are Basal-Like and Non-Basal-Like Triple-Negative Breast Cancers Really Different?

**DOI:** 10.1155/2020/4061063

**Published:** 2020-03-16

**Authors:** Atika Dogra, Anurag Mehta, Dinesh Chandra Doval

**Affiliations:** ^1^Department of Research, Rajiv Gandhi Cancer Institute and Research Centre, Rohini, Delhi 110085, India; ^2^Department of Laboratory Services, Rajiv Gandhi Cancer Institute and Research Centre, Rohini, Delhi 110085, India; ^3^Department of Medical Oncology, Rajiv Gandhi Cancer Institute and Research Centre, Rohini, Delhi 110085, India

## Abstract

**Objective:**

Triple-negative breast cancer (TNBC) accounts for 15–25% of breast cancers. It is increasingly recognized that TNBC is a motley disease. TNBC and basal-like (BL) subtype are different molecular classes of breast cancer with a high degree of overlap. However, a smaller fraction lacks the expression of basal markers in spite of being TNBC and is called non-basal-like (NBL). The aim of this study is to assess the clinicopathological features in TNBC and compare its BL and NBL subtypes. *Material and Methods*. A total of 200 subjects fulfilling the inclusion criteria of study were identified from the electronic medical records of institution. The tumor sections of subjects were immunohistochemically stained for basal markers, namely, 34*β*E12, c-Kit, and EGFR, in order to differentiate between BL and NBL subtypes. Comprehensive data were assembled from subjects' clinical records. The features of TNBC and their associations with the two subtypes were assessed using statistical analyses.

**Results:**

TNBC constituted 22% of all breast cancers. The family history of cancer was observed to be significantly associated with stage (*p*=0.013). The proportions of BL and NBL subtypes were equal. Of all parameters compared between two subtypes, only lymphovascular invasion was found to have statistically significant difference (*p*=0.019). Though no statistical significant difference between overall survival (OS) and disease-free survival (DFS) of two subgroups was found, BL subtype has slightly shorter DFS and OS compared to NBL.

**Conclusion:**

Both BL and NBL subtypes occur in equal proportions; hence, basalness and triple negativity are not synonyms. Though BL and NBL are prognostically similar, BL subtype shows a trend towards slightly shorter DFS and OS compared to NBL.

## 1. Introduction

Invasive breast carcinoma is the most commonly diagnosed cancer and the leading cause of cancer death among females worldwide [1]. Breast cancer is the topmost malignancy among Indian females with an annual incidence rate of 24.7 per million women [2]. It accounts for 15.46% of all cancers and 12.11% of cancer deaths in Indian population [2].

Breast cancer is a heterogeneous disease which is subdivided into many different entities, each having own clinical features and prognostic outcomes. Triple-negative breast cancer (TNBC) is clinically defined by the lack of expression of estrogen receptor (ER), progesterone receptor (PR), and lack of overexpression/amplification of human epidermal growth factor receptor-2 (HER2) proteins or HER2 gene copies. It accounts for 15–25% of newly diagnosed breast cancer cases [3]. It is widely considered to have aggressive clinical behavior, poor patient survival, and lack of targeted therapeutic option. This subgroup is one of the most challenging groups of breast cancers to treat. TNBC contributes to a large proportion of breast cancer deaths despite its small proportion among all breast cancers. Prevalence of TNBC in India is considerably higher compared with Western populations [4, 5]. The recent literature reports an exceptionally high frequency of TNBC (73.9%) in Indian premenopausal women below 35 years of age, in the prime of their reproductive life [6].

It is increasingly recognized that TNBC is a motley disease. The molecular subtypes include two basal-like (BL1 and BL2), an immunomodulatory, a mesenchymal, a mesenchymal stem-like, and a luminal androgen receptor subtypes [7]. TNBC and basal-like breast cancer (BLBC) are different molecular classes of breast cancer with a high degree of overlap. The majority of TNBC shows the expression of basal markers and many accept TNBC as BLBC. However, a smaller fraction lacks the expression of basal markers in spite of being TNBC and is called non-basal-like (NBL). The existing literature consistently indicates that basal-like (BL) subtype of TNBC shows a more aggressive behavior with poor prognosis. There is the scarcity of reliable data in the Indian setting and the aim of this study is to bridge this gap. More specifically, the aim of this study is to assess the clinicopathological parameters in TNBCs and compare these characteristics along with treatment outcome between BL and NBL subtypes of TNBC. In our preliminary study, a small group of TNBC was studied [8]. However; the current study includes a larger sample size and may be able to portray the clinical behaviors of BL and NBL subtypes of TNBC better.

## 2. Materials and Methods

### 2.1. Case Selection

The study was granted ethical approval by the Institutional Review Board. The medical records of total 3061 patients registered during January 2012 to May 2014 were screened. A number of 1579 cases who underwent complete biomarker testing (ER, PR, and HER2 overexpression/amplification) were studied of which 347 cases were found to be TNBC. A total of 200 subjects befitting the inclusion criteria of study were selected for the study. The patient selection criteria included the cases of TNBC having adequate material (blocks and clinical records) and treatment received at our institution. The detailed data regarding patients' clinical history, tumor characteristics, therapy, and recurrence, and so on were assembled from their clinical records as per the proforma of study. The entire group of patients was followed up periodically until October 2018. The follow-up information was gathered either by reviewing patients' clinical charts or through the telephonic interview.

### 2.2. Immunohistochemistry

The immunostaining was performed using formalin-fixed paraffin-embedded tissue sections. The sections were immunohistochemically stained for ER, PR, HER2, high molecular weight cytokeratins (HMWCKs), c-Kit, and EGFR (Table 1) according to the protocols provided by the manufacturer on automated immunostainer Ventana Benchmark XT (Roche/Ventana, Tucson, AZ, USA). The staining was done using 4 *μ*m sections. The heat induced antigen retrieval was done using CC1 at pH 8.4. Multimer-based strategy was used for labeling with ultraView detection kit. The staining of ER and PR was interpreted manually in accordance with Allred scoring and was considered positive only when >1% of tumor cells confirmed nuclear staining. HER2 immunohistochemical analysis was performed using the pathway FDA-approved test kit according to the manufacturer's instructions. The results were interpreted manually in accordance with the American Society of Clinical Oncology (ASCO)/College of American Pathologists (CAP) guidelines 2007 and subsequently from October 2013 in accordance with 2013 guidelines [9, 10]. The tumors with score 2+ were reassessed for amplification by fluorescence in situ hybridization and were labeled positive in accordance with the ASCO/CAP guidelines (2007 and 2013) as mentioned ibid [9, 10].

**Figure 1:**
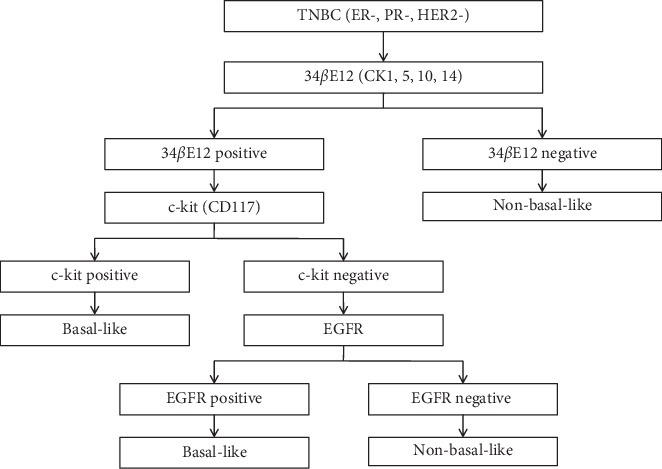
Algorithm for defining basal-like breast cancer (adopted from Nielsen et al.).

**Figure 2:**
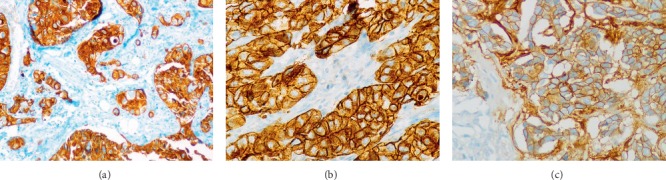
Positive immunohistochemical staining for (a) 34*β*E12; 40x × 10x, (b) c-Kit; 40x × 10x, and (c) EGFR; 40x × 10x.

The algorithm used for separating BL and NBL subtypes (Figure 1) has been adopted from Nielson et al. [11] and the same was followed in our preliminary study [8]. The IHC for 34*β*E12 was performed first to identify the expression of basal markers and the presence of HMWCKs (CK5, 6, 10, and 14) was confirmed. The pattern of cytoplasmic stain was considered as positive staining (Figure 2). The IHC for c-Kit (CD117) was carried out after getting positive staining result for 34*β*E12.

The presence of cytoplasmic staining along with membranous staining was regarded as positive staining for c-Kit (Figure 2). Further, IHC for EGFR was performed to assure the expression on basal markers in only those cases in which tumor cells reveal positive staining for 34*β*E12 and negative staining for c-Kit. The presence of cytoplasmic staining along with membranous staining was considered as positive staining for EGFR (Figure 2). In conclusion, the TNBC cases show staining patterns either 34*β*E12+/c-Kit + or 34*β*E12+/c-Kit-/EGFR+ were categorized as BL and the rest as NBL (Figure 1). To observe if the correct staining procedure was followed, a positive control from the tissue known to contain the antigen under examination was kept on each slide.

### 2.3. Statistical Analysis

The statistical analyses were performed using IBM SPSS software (Version 23, SPSS Inc, Chicago, IL, USA). The qualitative data were presented in frequencies/proportions and quantitative data were presented by the mean (standard deviation [SD]) or median (range). The subjects with missing information were excluded from the analysis. To calculate the statistical significance, Pearson Chi-square and Fisher's exact tests were applied for categorical variables; however, independent-samples *t*-test was used for the analysis of continuous versus categorical variable. The overall survival (OS) was calculated as the duration between the date of diagnosis and date of last contact/death. The disease-free survival (DFS) was measured from the date of surgery until date of relapse/progression of disease/last contact. In nonoperated cases, the DFS was calculated from the date of chemotherapy (CT) completion to the date of relapse/progression of disease/last contact. The log-rank test was applied to compare Kaplan–Meier curves for survival analysis.

## 3. Results

A total of 200 cases were selected for this prospective study. Of all registered cases to the institute during the mentioned period, approximately half of the proportion (51.6%) had undergone testing for ER, PR, and HER2. Of all tested, 22% (347/1579) cases were TNBC.  Table 2 summarizes the background data including clinical and pathological features of these cases. The mean (SD) and median (range) ages of our study population were 49.7 (11.9) and 48.5 (59) years, respectively. The most common age at menarche was between 12– 15 years and age at first childbirth was between 21 and 25 years. The most common duration of noticing the lump first time and seeking medical advice was 1–6 months (44.5%). While a good proportion of women sought for medical advice within a month (35.8%), a few delayed it for more than 6 or 12 months (19.7%). The family history of breast/ovarian cancer was positive in around 13% (19/149) cases.

Clinical stage II was predominant in our study cohort and was followed by stages III, I, and IV (Table 2). The most common tumor size was between 2-3 cm (34%) followed by 3–4 cm (28%). Among 23 cases with T4 stage, peau d'orange was found in 15 (65.2%) cases; however, inflammation/dimpling/fungating mass was present in 4 (17.4%) cases and the rest (17.4%) had no skin changes. A total of 189 (94.5%) patients had undergone surgery. Almost all patients (98%) were advised with CT. The most common CT regime given was PACS 01 (3 cycles of fluorouracil, epirubicin, and cyclophosphamide followed by 3 cycles of docetaxel). Largely, the type of treatment given was the combination of all three modalities, that is, surgery, CT, and radiotherapy. The recurrence of disease took place in 16% (32/200) of the cases and primarily in the form of distant metastasis. Lung was the most common site of metastasis followed by brain. The occurrence of second primary was observed in only 2% (4/200) of the patients. At a median follow-up of 58 months, 19.5% (39/200) of the patients died, of which 16.5% were disease-related deaths.

The IHC evaluation revealed that 50.5% (101/200) of TNBC were BL and 49.5% (99/100) were NBL phenotype. The clinical and pathological parameters were compared to assess the difference between two groups (Table 3). Of all parameters, only LVI was found to have a statistically significant difference (*p*=0.019) between the two phenotypes of TNBC and was associated with NBL subtype (Table 3). The family history of cancer was also evaluated to find the association with clinicopathological factors (Table 4). Early stage was found to be significantly associated with the positive family history of cancer (*p*=0.013). The time interval from the first symptom to diagnosis was significantly related to stage group (*p* < 0.001) and the vital status (*p*=0.022) as revealed in Table 5.

**Figure 3:**
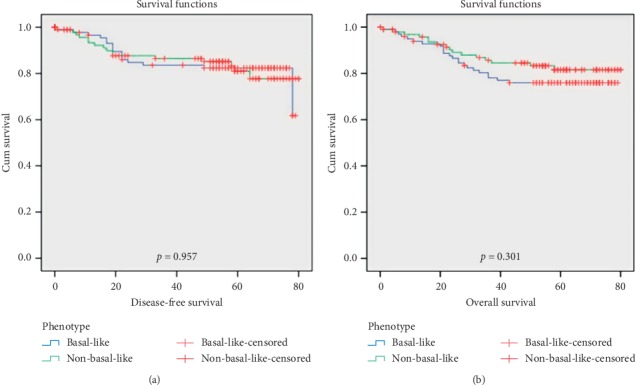
Kaplan–Meier curves for (a) disease-free survival and (b) overall survival.

The mean and median follow-up durations in our group population were 51 and 58 months, respectively. The mean DFS of the entire study cohort was 48 months. The recurrence occurred in 16% (32) of the cases. Both subgroups of TNBC showed an equal number of relapses. At 6.5 years, the DFS of BL group was 62% and that of NBL was 78% (Figure 3). The average OS of entire group was found to be 51 months with 19.5% deaths. A total of 23% patients died in BL group; however, 16% of deaths were observed in NBL group (*p*=0.301). The BL and NBL groups had 76% and 82% OS, respectively, at 6.5 years (Figure 3). The analyses were done to find out the difference in survivals (OS and DFS) between BL and NBL biological subtypes. There was no statistically significant difference between OS and DFS of the two subgroups despite having inferior survivals in BL cluster.

## 4. Discussion

There is a paucity of Indian literature on BL subtype of TNBC which necessitates studying this group due to its aggressive clinical course and grim outcomes. We found 22% of total breast cancer cases as TNBC which has a similar incidence to that reported in some Indian studies [12, 13]. However, many Indian studies have reported higher TNBC prevalence such as 39.8% and 43.5% [5, 14]. Thakur et al. [15] have suggested that India ranks the top in the world in incidence and prevalence of TNBC. Our study includes 200 cases of TNBC with a mean age of 49.7 years. This may be considered as younger age and is comparable to other Indian studies [4, 6]. In contrast to this, older age at diagnosis was observed in Western studies [16, 17]. This shows that TNBC is affecting young Indian population [18] and most probably reflects the general tendency of breast cancers occurring a decade earlier. Despite being at a young age, the majority of patients considered in this study were postmenopausal. This is in consistent with the findings that the average age of menopause of an Indian woman is much less (46.2 years) than their Western counterparts (51 years) [18]. In addition, it may be due to some other biological differences between both populations which are unknown as yet.

Clinically, stage II disease is followed by III, I, and IV which is in accordance with previous findings [8]. Also, this is related to a large proportion of cases that underwent surgery in our study group which was considered the primary treatment for breast cancer during the analyzed period. But according to the recent guidelines, a neoadjuvant approach should be preferred in subtypes highly sensitive to chemotherapy such as triple-negative breast cancer [19]. The proportion of family history of any cancer in our study was in accordance with that by Mori et al., 2018 [20]. Similarly, the family history of breast/ovarian cancer was comparable with a Pakistani study [21]. The family histories of any cancer and breast/ovarian cancer were significantly associated with early stages (I and II) of TNBC which reflects the awareness about the disease in family members/relatives of cancer patients and their promptness in reporting to a clinic for examination and medical advice.

The proportions of BL (50.5%) and NBL (49.5%) were approximately equal in this study group which is in congruence to the findings [17]. By and large, others have reported a higher proportion of BL compared to NBL [22, 23]. This may indicate the uniqueness of TNBC in Indian women. Also, it reveals that the terms TNBC and BLBC are not synonyms [23, 24]. In the present study, except for LVI, no statistically significant association could be seen between clinicopathological and TNBC subtypes. Surprisingly, the presence of LVI was significantly associated with NBL cluster [25]. It reveals that NBL may not be a less aggressive phenotype than BL. The longer time interval from the first symptom to diagnosis was significantly related to the advance stage, large tumor size, and high frequency of mortality [26, 27]. It shows that the diagnostic delay is a significant problem and there is a need to improve breast cancer awareness among the population.

At a median follow-up of 58 months, the recurrence occurred in 16% cases with the lung being the most common site followed by the brain [28, 29]. The rarity of local recurrence and high rates of distant recurrence in our study suggest that TNBC has a propensity to develop visceral metastasis during the course of disease [30, 31]. At 6.5 years, the DFS of BL and NBL group was 62% and 78%, respectively, without any significant difference. Though the number of cases having disease recurrence was equal in both subgroups, still the DFS in BL group was lower than that in NBL. The OS in the BL group was inferior compared to the NBL cluster, but the difference was not statistically significant.

Though not many studies have been undertaken in India on this subject, ours is the first research study that compares the clinicopathological profiles and survival outcomes between BL and NBL subtypes in a large sample of TNBC. It may contribute to untangling of the mystery of TNBC in the Indian population.

The potential weakness of our study is unavoidable selection biases for the sake of tumor tissue. However, it doesn't affect the staging statistics much, as previous studies from our institution have also reported stage II as the predominant stage in breast cancer cases [8].

## 5. Conclusion

In conclusion, TNBC may affect one in four women with breast cancer. Both BL and NBL subtypes occur in equal proportions. TNBC and basalness are not synonyms. LVI is the only parameter that significantly differentiates BL form NBL subtype. Though both TNBC subgroups are prognostically similar, BL subtype shows a trend towards slightly shorter DFS and OS compared to NBL. Further research to unravel the molecular heterogeneity of TNBC for the development of targeted therapies is warranted.

## Figures and Tables

**Table 1:** Specifications of antibodies used.

Marker	Clone	Manufacturer	Dilution	Pattern of staining		
				*Nuclear*	*Cytoplasm*	*Membranous*
ER	SP1	Ventana	RTU	+	NA	NA
PR	1E2	Ventana	RTU	+	NA	NA
HER2	4B5	Ventana	RTU	NA		+
High molecular weight cytokeratins	34*β*E12	Dako	1 : 50	NA	+	NA
c-Kit (CD 117)	Polyclonal	Dako	1 : 400	NA	+	+
EGFR	EP774Y	Biocare	RTU	NA	+	+

*∗*ER, estrogen receptor; PR, progesterone receptor; EGFR, epidermal growth factor receptor; RTU, ready to use; NA, not applicable.

**Table 2:** Patient and tumor characteristics of triple-negative breast cancer.

Characteristic (*N*)	Frequency (%)
Age (years) (200)	
Mean (SD)	49.7 (11.9)
Median (range)	48.5 (59)
Menstrual history (200)	
Premenopausal	81 (40.5)
Perimenopausal	16 (8)
Postmenopausal	103 (51.5)
Marital status	
Married	197 (98.5)
Unmarried	3 (1.5)
Age at first childbirth (years) (99)	
15–20	14 (14.1)
21–25	51 (51.5)
26–30	22 (22.2)
31–35	1 (1)
Not yet	11 (11.1)
Parity (150)	
Not yet	9 (6)
1	19 (12.7)
2	57 (38)
3	43 (28.7)
4 or more	22 (14.6)
Family history of cancer (149)	
Yes	34 (22.8)
No	115 (77.2)
Laterality (200)	
Right	109 (54.5)
Left	91 (45.5)
Tumor location (200)	
Upper outer quadrant	103 (51.5)
Upper inner quadrant	36 (18)
Lower outer quadrant	19 (9.5)
Lower inner quadrant	7 (3.5)
Central	26 (13)
Multiple quadrants	8 (4)
Axillary tail	1 (0.5)
Stage (200)	
I	11 (5.5)
II	136 (68)
III	43 (21.5)
IV	10 (5)
Surgical procedure (189)	
MRM	121 (64)
BCS	68 (36)
Primary histology (200)	
IDC	190 (95)
Metaplastic	6 (3)
Others	4 (2)
Grade (200)	
II	27 (13.5)
III	173 (86.5)
Lymph node status (189)	
Positive	86 (45.5)
Negative	103 (54.5)
Extracapsular extension (86)	
Present	58 (67.4)
Absent	28 (32.6)
Lymphovascular invasion (194)	
Present	81 (41.8)
Absent	113 (58.2)
Subtype (200)	
Basal-like	101 (50.5)
Non-basal-like	99 (49.5)
Recurrence (200)	
No	168 (84)
Local	2 (1)
Locoregional	1 (0.5)
Distant	29 (14.5)
Site of metastasis (29)	
Lung	7 (24.2)
Liver	5 (17.2)
Brain	4 (13.8)
Others	13 (44.8)
Vital status (200)	
Alive	161 (80.5)
Dead	39 (19.5)
Cause of death (39)	
Disease-related	33 (84.6)
Non-disease-related	6 (15.4)

MRM, modified radical mastectomy; BCS, breast conservative surgery; IDC, infiltrating ductal carcinoma; SD, standard deviation.

**Table 3:** Comparison between basal-like and non-basal-like subtypes.

Characteristic (*N*)	Basal-like *N* (%)	Non-basal-like *N* (%)	Chi-square	*p* value
Menstrual history (200)				
Premenopausal	44 (43.6)	37 (37.4)	0.922	0.631
Perimenopausal	7 (6.9)	9 (9.1)		
Postmenopausal	50 (49.5)	53 (53.5)		
Age at first childbirth (years) (99)				
15–20	5 (11.1)	9 (16.7)	6.189	0.185
21–25	27 (60)	24 (44.4)		
26–30	10 (22.2)	12 (22.2)		
31–35	1 (2.2)	0 (0)		
Not yet	2 (4.4)	9 (16.7)		
Parity (150)				
1	12 (16.2)	7 (9.2)	8.285	0.141
2	25 (33.8)	32 (42.1)		
3	21 (28.4)	22 (28.9)		
4 or more	14 (18.9)	8 (10.5)		
Not yet	2 (2.7)	7 (9.2)		
Family history of cancer (149)				
Yes	18 (24)	16 (21.6)	0.120	0.729
No	57 (76)	58 (78.4)		
Stage (200)				
I	3 (3)	8 (8.1)	3.906	0.272
II	69 (68.3)	67 (67.7)		
III	22 (21.8)	21 (21.2)		
IV	7 (6.9)	3 (3)		
Grade (200)				
II	11 (10.9)	16 (16.2)	0.306	0.189
III	90 (89.1)	83 (83.8)		
Lymph node status (189)				
Positive	43 (46.7)	43 (44.3)	0.111	0.740
Negative	49 (53.3)	54 (55.7)		
Extracapsular extension (86)				
Present	26 (60.5)	32 (74.4)	1.906	0.167
Absent	17 (39.5)	11 (25.6)		
Lymphovascular invasion (194)				
Present	35 (34.7)	46 (46.5)	7.908	**0.019** *∗*
Absent	60 (59.4)	53 (53.5)		
Recurrence type (32)				
Local	1 (6.3)	1 (6.3)	1.034	0.596
Locoregional	0 (0)	1 (6.3)		
Distant	15 (93.8)	14 (87.5)		
Vital status (200)				
Alive	78 (77.2)	83 (83.8)	1.392	0.238
Death	23 (22.8)	16 (16.2)		
Cause of death (39)				
Disease-related	21 (91.3)	12 (75)	1.927	
Non-disease-related	2 (8.7)	4 (25)		0.165

*∗p* < 0.05.

**Table 4:** Family history of cancer and clinicopathological factors.

Characteristic (*N*)	With family history *N* (%)	Without family history *N* (%)	Chi-square	*p* value
Stage (149)				
I	4 (11.8)	5 (4.3)	10.729	**0.013** *∗*
II	27 (79.4)	79 (68.8)		
III	1 (2.9)	26 (22.6)		
IV	2 (5.9)	5 (4.3)		
Grade (149)				
II	2 (5.9)	15 (13)	1.331	0.249
III	32 (94.1)	100 (87)		
Lymph node status (141)				
Positive	12 (37.5)	50 (45.9)	0.704	0.402
Negative	20 (62.5)	59 (54.1)		
Extracapsular extension (62)				
Present	5 (41.7)	34 (68)	2.876	0.088
Absent	7 (58.3)	16 (32)		
Lymphovascular invasion (144)				
Present	11 (34.4)	46 (41.1)	1.325	0.516
Absent	21 (65.6)	66 (58.9)		
Recurrence (149)				
Yes	4 (11.8)	16 (13.9)	0.104	0.999
No	30 (88.2)	99 (86.1)		
Vital status (149)				
Alive	29 (85.3)	96 (83.5)	0.064	0.999
Dead	5 (14.7)	19 (16.5)		

*∗p* < 0.05.

**Table 5:** Association of diagnostic delay and features.

Characteristic (*N*)	≤1 month	1.1–6 months	6–12 months	>12 months	Chi-square	*p* value
Stage (182)						
I-II	58 (89.2)	57 (70.4)	17 (68)	2 (18.2)	26.415	0.000*∗∗*
III-IV	7 (10.8)	24 (29.6)	8 (32)	9 (81.8)		
Grade (182)						
II	9 (13.8)	10 (12.3)	5 (20)	2 (18.2)	1.062	0.786
III	56 (86.2)	71 (87.7)	20 (80)	9 (81.8)		
Recurrence (182)						
Yes	12 (18.5)	12 (14.8)	3 (12)	1 (9.1)	1.048	0.790
No	53 (81.5)	14 (85.2)	22 (88)	10 (90.9)		
Vital status (182)						
Alive	55 (84.6)	66 (81.5)	21 (84)	5 (45.5)	9.646	**0.022** *∗*
Dead	10 (15.4)	15 (18.5)	4 (16)	6 (54.5)		

*∗p* < 0.05; *∗∗p* < 0.001.

## Data Availability

The data used to support the outcomes of the study are already incorporated in the article. Supplementary data may be made available upon request to the corresponding author as it is not being shared publicly in order to preserve the anonymity of our patients.
